# Organ‐Specific Microplastic Profiling and Polymer Characterization in Freshwater Fish Species of Karnataka

**DOI:** 10.1155/jt/8854199

**Published:** 2026-05-31

**Authors:** Kuruveetil Manikandan Ashitha, Anjali Tamrakar, Gopika Melethil, Anish Kumar Warrier

**Affiliations:** ^1^ Centre for Climate Studies, Manipal Institute of Technology, Manipal Academy of Higher Education, Manipal, 576104, Karnataka, India, manipal.edu; ^2^ School of Environmental Studies, Cochin University of Science and Technology, Kochi, India, cusat.ac.in

**Keywords:** freshwater fish, health, microplastics, multiorgan analysis, risk assessment

## Abstract

Microplastic (MP) contamination is an emerging threat to freshwater ecosystems and food security. The objective of this study was to provide a comprehensive baseline assessment of MP abundance, polymer composition, and tissue‐specific distribution across five organs (gut, gills, liver, gonads, and muscle) of two economically vital species *Labeo rohita* and *Pangasius pangasius*, in Udupi, Karnataka, India. MPs were detected in 100% of specimens, with higher mean (± standard deviation) abundances of 58.27 ± 10.48 particles per individual (particles ind^−1^) in *L. rohita* and 42.40 ± 5.40 particles ind^−1^ in *P. pangasius*. While *L. rohita* showed preferential accumulation in the gills and gut, in *P. pangasius*, MPs were more uniformly distributed across organs. Fibers dominated the assemblage (97.68%), with polypropylene and polyester identified as the primary polymers via Fourier transform infrared spectroscopy with attenuated total reflectance (FTIR–ATR) analysis. We conclude that the pervasive presence of MPs across all tissues, particularly the high accumulation in edible muscle and reproductive gonadal tissue, signals an urgent ecological risk and a direct pathway for human dietary exposure. The detection of MPs in gonadal tissues, while not yet linked to observed physiological impairment, identifies a potential reproductive risk and highlights a critical area for future histological and toxicological investigation. These findings establish a baseline for assessing the long‐term health of local fisheries.

## 1. Introduction

Plastic has become indispensable in modern life, with global production exceeding 400 million tons annually [1, 2]. Plastic debris has become ubiquitous in natural environments worldwide [3], and its persistence and fragmentation have raised increasing concern about potential impacts on wildlife and ecosystem health [4]. However, its excessive use and improper disposal have led to widespread environmental contamination. Once released into the environment, larger plastic items (macroplastics, typically > 25 mm) undergo physical, chemical, and biological degradation, fragmenting into smaller particles termed microplastics (MPs), a concept first introduced by Thompson [5] and later refined by Arthur [6] and Frias and Nash [7]. MPs occur in various forms, including fibers, fragments, films, pellets, and foams, depending on their origin and the processes governing their degradation [8–10]. MPs are commonly classified as (i) primary MPs, intentionally manufactured small particles such as microbeads and industrial pellets, and (ii) secondary MPs, derived from the breakdown of larger plastics, including packaging materials, fishing gear, and agricultural plastics under sunlight, abrasion, and mechanical forces [11, 12]. These particles are now ubiquitous across environmental matrices, including marine and freshwater ecosystems, sediments, soils, and even the atmosphere [13].

While marine MP pollution has received considerable global attention, freshwater environments remain comparatively understudied [14]. Freshwater bodies not only sustain aquatic biodiversity but also act as important intermediaries for the transport of plastics from terrestrial sources into aquatic environments and ultimately the oceans, contributing an estimated 1.15–2.41 million tons of plastic annually [15, 16]. MPs enter freshwater systems from a complex array of sources, including stormwater runoff, wastewater effluents, agricultural activities, tire wear, domestic laundering, and atmospheric deposition. Their distribution is strongly influenced by watershed characteristics, land use patterns, socioeconomic factors, and waste management practices [2, 14].

Once ingested or inhaled by aquatic organisms, MPs may accumulate in tissues, adhere to gill surfaces, or translocate to the circulatory system [17]. Such internalization can impair feeding behavior, immune responses, and reproduction and induce oxidative stress [18]. MPs also have a strong affinity for contaminants, including heavy metals and hydrophobic organic pollutants, and often accumulate at concentrations several times higher than those in the surrounding water [19, 20]. These polluted particles may be transferred through food webs, ultimately posing health risks to higher trophic levels and humans [14, 21, 22]. Recent studies have detected MPs in human tissues, such as the gastrointestinal tract, placenta, and breast milk [2, 23, 24], with long‐term exposure linked to oxidative stress, cytotoxicity, metabolic disorders, and immune dysfunction [15, 25].

Fish play a pivotal ecological and economic role in freshwater ecosystems and serve as a major source of dietary protein for millions of people. Despite India being the world’s second‐largest fish producer, with inland fisheries in states such as Karnataka contributing substantially to food security [26, 27], comprehensive assessments of MP internal loading remain limited. Most existing studies focus primarily on the gastrointestinal tract, which is typically discarded prior to consumption, thereby potentially underestimating the actual dietary exposure risk associated with MPs accumulated in edible muscle tissue.

Furthermore, although the respiratory and digestive pathways of MP uptake are well documented, the systemic translocation of these particles to vital metabolic and reproductive organs, such as the liver and gonads, is a critical yet underexplored frontier in aquatic toxicology. The Udupi region, a key hub for inland fisheries in Karnataka, offers a unique case study owing to its high population density and diverse anthropogenic inputs; however, baseline data on MP contamination in its freshwater species are currently lacking [28].

The significance of this study lies in its multiorgan approach. By examining MP distribution across five distinct tissues (gut, gills, muscle, liver, and gonads) in *Labeo rohita* and *Pangasius pangasius*, this study advances beyond simple presence–absence assessments. In particular, it addresses the critical “blind spot” of reproductive organ contamination, providing evidence of how MP exposure may impact the long‐term sustainability of freshwater fisheries and pose risks to human health through trophic transfer. This study addresses existing knowledge gaps by investigating MP contamination in two widely consumed freshwater fish species, *L. rohita* (rohu) and *P. pangasius* (panga), obtained from local markets in Udupi, Karnataka. These species were selected due to their commercial importance, high dietary relevance, and direct linkage to human exposure pathways [12, 25]. These findings align with the United Nations Sustainable Development Goals SDG 3 (Good Health and Well‐Being) and SDG 14 (Life Below Water) by providing the empirical baseline necessary for developing targeted pollution mitigation and food safety protocols in freshwater ecosystems.

## 2. Materials and Methods

### 2.1. Sample Collection

To establish a robust and representative baseline, two freshwater fish species of high dietary and commercial importance were procured from local markets in Udupi, Karnataka, during December 2024, reflecting prevailing consumption patterns along the southwest coast of India [29]. A total of 30 specimens were collected, comprising *L. rohita* (rohu; family Cyprinidae; *n* = 15) and *P. pangasius* (panga; family Pangasiidae; *n* = 15). The mean total length and weight were 41.63 ± 1.55 cm and 0.86 ± 0.10 kg for *L. rohita* and 42.37 ± 2.14 cm and 1.00 ± 0.15 kg for *P. pangasius*, respectively. Five organs (gills, gut, liver, gonads, and muscle) from each fish were dissected and analyzed for MP content. This organ‐resolved sampling design enabled a high‐resolution assessment of both intra‐ and interspecific distribution of MPs.

Specimens were procured in multiple batches to capture natural variability, transported in insulated containers to the laboratory within 2 h, under controlled conditions to prevent contamination, and stored at −20°C until analysis. The number of specimens was constrained by the limited availability of freshly landed, locally sourced freshwater fish, as the region’s fisheries and market demand are predominantly for marine fish. Although several studies have focused on marine seafood, research on freshwater fish often consumed as a delicacy remains scarce. Fresh individuals of comparable size and physiological condition were intermittently available due to fluctuations in seasonal catch and market supply [30–32].

While procurement in multiple batches helped to incorporate natural variability, the overall sample size (*n* = 15 per species) remains a limitation when interpreting broader regional patterns and population‐level variability. Therefore, the findings presented here should be viewed as a baseline dataset that highlights the occurrence of MPs in two widely consumed freshwater species, and future studies with larger sample sizes and broader spatial coverage are recommended to strengthen statistical power and generalizability.

### 2.2. Sample Preparation and Extraction of MPs

Frozen fish specimens were thawed at room temperature prior to analysis and gently rinsed with prefiltered Milli‐Q water to remove surface debris and minimize external contamination of the samples. Morphometric parameters, including total length, body weight (BW), and mouth dimensions, were recorded using calibrated Vernier calipers (Supporting Table S1). All instruments were cleaned with 70% ethanol and prefiltered Milli‐Q water prior to use. Laboratory procedures were performed under contamination‐controlled conditions: Glassware was covered when not in use, cotton laboratory coats were worn, and procedural blanks were included to monitor the potential airborne contamination. Each fish was dissected to isolate the five target organs (gut, gills, muscle, liver, and gonads), which were transferred to precleaned, covered glass beakers. Organs were weighed (wet weight [WW]) to the nearest 0.01 g [33–35]. Digestion was carried out using 300 mL of 10% (w/v) KOH, with samples incubated at 60°C for 48 h [36]. For tissues with high lipid or organic content, an additional 20 mL of 30% H_2_O_2_ was introduced following the initial KOH digestion, and samples were further incubated for 24 h at room temperature to ensure complete removal of organic matter. The resulting digests were vacuum‐filtered through Whatman Grade 1 cellulose filter papers (47 mm diameter; 11 μm pore size) to maintain consistency across all samples. This setup captured MPs 11 μm and larger, allowing reliable detection and characterization of particles within this size range. To minimize particle loss, filtration units and funnels were prerinsed with prefiltered Milli‐Q water, and the filters were handled exclusively using stainless steel forceps. The retained residues were air‐dried in covered glass Petri dishes and stored in desiccators until they were examined microscopically.

### 2.3. Identification of MPs

The filtered residues were examined under a Zeiss Stemi 508 stereomicroscope and photographed using an Axiocam 208 color camera at 80× magnification. Putative MP particles were initially screened based on established visual criteria, including morphology, color uniformity, homogeneity, and the absence of cellular or organic structures. To minimize the misclassification of natural fibers or tissue remnants, Rose Bengal staining was applied, which selectively colors biological material and aids in distinguishing synthetic particles from natural debris [11, 13, 37].

Polymer composition was identified using Fourier transform infrared spectroscopy with attenuated total reflectance (FTIR–ATR) across a spectral range of 400–4000 cm^−1^ at a resolution of 4 cm^−1^ with 32 co‐added scans [38–40]. Spectra were processed using Open specy; only matches with a match value > 0.80 were accepted as confirmed polymers [3]. Complementary morphological and elemental assessments were performed using scanning electron microscopy (SEM) coupled with energy‐dispersive spectroscopy (EDS). SEM provided high‐resolution visualization of surface textures and degradation features, whereas EDS offered semiquantitative elemental profiles that helped differentiate synthetic polymers from mineral particulates. All confirmed MPs were categorized by shape (fibers, fragments, films, pellets, and foams), size (11–5000 μm), and color (blue, black, green, transparent, red, and others).

### 2.4. Quality Control and Recovery Assessment

All procedures, from sample thawing and dissection to digestion and identification, were conducted under controlled laboratory conditions to prevent contamination [18, 39, 41]. Milli‐Q ultrapure water was used exclusively for cleaning glassware, preparing solutions, and rinsing equipment. Glassware and dissection instruments were washed with ethanol and covered with aluminum foil when not in use. To avoid procedural contamination, only glass and metal materials were used, and laboratory personnel wore nitrile gloves and cotton coats throughout the process.

To assess potential airborne and procedural contamination, two types of blanks were employed: (1) atmospheric blanks (open Petri dishes) and (2) liquid procedural blanks (reagents processed without tissue but subjected to identical analytical procedures). One procedural blank was included for every batch of 10 samples. No MPs were detected in any of the blanks, indicating negligible background contamination [23, 42, 43].

The recovery efficiency was evaluated by spiking representative tissue samples with known quantities of polyethene and polypropylene MP standards (fibers and fragments). The recovery rate exceeded 95%, consistent with previously published values, confirming the reliability of the extraction protocol.

### 2.5. Risk Assessment

Several environmental and exposure‐based indices were used to evaluate the ecological and human health risks associated with MP contamination.

#### 2.5.1. Coefficient of Microplastic Impact (CMPI)

CMPI quantifies the relative contribution of each MP shape to the total MP load [44, 45]. This index provides insights into morphology‐specific risks, particularly when fiber‐dominated MP assemblages may pose enhanced biological stress. The index was calculated as follows:

(1) CMPI=number of MPs of a specific shapetotal number of MPs.

#### 2.5.2. Polymer Hazard Index (PHI)

The PHI evaluates the toxicity potential associated with identified polymers based on the hazard scores assigned to individual polymer types [46]. The PHI was calculated as follows:

(2) PHI=∑Sn×Pn,

where *S*_*n*_ represents the percentage of each polymer type and *P*_*n*_ denotes the corresponding hazard score. The PHI categories ranged from low (0.1) to extreme hazard (> 1000).

#### 2.5.3. Estimated Daily Intake (EDI) and Estimated Annual Intake (EAI)

Human exposure to MPs via fish consumption was estimated using ingestion‐based metrics, as described by Makhdoumi [47]. The EDI (particles/kg‐bw/day) was calculated as follows:

(3) EDI=IR×CBW,

where *C* is the concentration of MPs in the fish tissue, IR is the ingestion rate, and BW is body weight.

Long‐term exposure was assessed using the EAI (particles/kg‐bw/year):

(4) EAI=EDI×365.

To normalize the exposure by BW, the EAI values were divided by the average adult BW of 60 kg. The ingestion rate was based on Karnataka’s per capita fish consumption of 11.6 kg/year (31.8 g/day), as reported by the Ministry of Fisheries, Animal Husbandry, and Dairying [26, 48].

### 2.6. Data Analysis

The MP abundance for each species was calculated as the mean number of MPs per individual, with corresponding standard deviations. Identified particles were categorized by size, shape, and color, and their relative proportions were expressed as percentages. Statistical analyses were performed using Microsoft Excel (2019). The Shapiro–Wilk test was applied to assess the normality of the MP concentration data, ensuring suitability for parametric comparisons.

## 3. Results and Discussion

### 3.1. Fish Species

The distinct ecological niches and feeding strategies of *L. rohita* and *P. pangasius* likely govern their differential MP profiles. As a column‐to‐benthic herbivore, *L. rohita* primarily feeds on periphyton and detritus and may inadvertently trap suspended microfibers, which are the dominant morphology identified in this study [27, 49, 50]. This “filtering” effect may explain the higher mean MP abundance observed in *L. rohita* (58.27 ± 10.48 particles ind^−1^) compared to the omnivorous *P. pangasius* (42.40 ± 5.40 particles ind^−1^), which predominantly consumes larger macrofauna. To ensure statistical comparability, sampled cohorts were restricted to a narrow morphometric range (38–45 cm length; 0.5–1.23 kg weight), thereby minimizing ontogenetic variations in ingestion rates.

While direct environmental sampling provides site‐specific insights, market‐based sampling offers a more representative estimate of human dietary exposure from the regional food supply. A synthesis of previous studies on MPs in *L. rohita*, *P. pangasius*, and related freshwater species, including information on target organs, particle characteristics, and polymer types, is presented in Supporting Table S2. By expanding the analytical scope to include reproductive tissues, a commonly overlooked “blind spot” in the current literature, this study advances beyond presence–absence assessments to provide a comprehensive evaluation of physiological and food safety risks associated with freshwater contamination.

### 3.2. MP Occurrence in Organs

MPs were detected across all examined organs, indicating systemic contamination within the freshwater ichthyofauna of the Udupi region. The pooled distribution (*n* = 1510 particles) followed the gradient: gut (*n* = 359) > gills (*n* = 357) > gonads (*n* = 280) > liver (*n* = 260) > muscle tissue (*n* = 254). This hierarchy supports a dual‐pathway exposure model, wherein ingestion constitutes the primary route, while the branchial (gill) surface acts as a critical secondary interface for the entrapment of suspended fibers [13, 51]. The observed distribution reflects a combination of biological controls, such as feeding behavior and habitat preference, and environmental factors, including ambient MP concentrations and hydrodynamic conditions [19, 52, 53].

**FIGURE 1:**
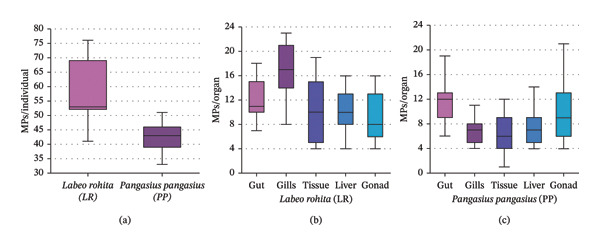
Boxplots of microplastic (MP) abundance in *Labeo rohita* (LR) and *Pangasius pangasius* (PP): (a) per individual; (b, c) organ‐wise concentrations (gut, gills, muscle, liver, and gonads). The boxes show the median, IQR, and outliers.

In *L. rohita*, the mean abundance of MPs was 58.27 ± 10.48 particles ind^−1^ (Figure 1), with gills emerging as the dominant accumulation site (16.53 ± 4.56 particles ind^−1^), diverging from the predominantly gastrointestinal‐focused patterns reported in earlier studies [54, 55]. As a column‐to‐benthic feeder, *L. rohita* continuously processes large volumes of water and detritus. The extensive surface area and mucus‐rich epithelium of the gills likely facilitate passive adsorption of microfibers from the water column, representing a continuous “inhalation‐like” exposure pathway [56, 57].

In contrast, *P. pangasius* exhibited a more ingestion‐dominant profile, with the highest MP load recorded in the gut (11.67 ± 4.47 particles ind^−1^). Notably, despite its higher trophic position as an omnivore, *P. pangasius* displayed lower overall MP loads than the herbivorous *L. rohita*. This suggests that, within these habitats, exposure pathways linked to habitat use (surface/column vs. deeper waters) and mechanical filtration by gill structures exert a stronger influence on MP accumulation than trophic magnification alone [38, 58].

The mean MP abundance was lower than that in *L. rohita*, at 42.40 ± 5.40 particles ind^−1^ (Figure 1). In this species, the gut exhibited the highest MP load (11.67 ± 4.47 particles ind^−1^), whereas the muscle tissue had the lowest load (6.67 ± 4.09 particles ind^−1^). This distribution is more typical of ingested MPs, where particles are primarily accumulated in the gastrointestinal tract, and only a fraction, potentially the smaller ones, may translocate to internal tissues. Interestingly, although omnivorous or carnivorous species are generally expected to ingest higher amounts of MPs due to their broader diets and higher trophic positions [54], the present study recorded a greater MP burden in the herbivorous *L. rohita* than in *P. pangasius*. This observation is consistent with the findings of Patidar [45], who also reported comparatively elevated MP loads in *L. rohita* relative to other freshwater species. A plausible explanation is that herbivorous fish feeding on periphyton, macrophytes, and detritus are particularly susceptible to MPs that have settled onto substrates, become entrapped within biofilms, or adhere to plant surfaces, thereby increasing the likelihood of incidental ingestion.

Statistical analysis using the Shapiro–Wilk test showed that MP concentrations (expressed as MPs per gram of tissue) did not deviate significantly from a normal distribution in either species (*p* > 0.05), indicating that parametric approaches could be appropriately used for further comparative analyses. Beyond mere abundance, the detection of MPs in internal organs such as the liver and gonads is particularly concerning [59]. Their presence in the liver, muscle, and gonads provides clear empirical evidence of systemic translocation. For particles to move from primary entry routes (gut and gills) to internal tissues, they must overcome biological barriers. Particles smaller than 150 μm, and especially those below 120 μm, can cross the intestinal epithelium via paracellular transport or persorption, subsequently entering the circulatory and lymphatic systems [24, 60].

The occurrence of MPs in the liver suggests transport through the hepatic portal system. As the central organ for detoxification, the liver’s high degree of vascularization makes it a likely sink for translocated particles, potentially leading to metabolic stress and oxidative imbalance [5, 61, 62]. The detection of MPs in gonadal tissue (3.68 ± 0.09 particles ind^−1^ in *L. rohita*) is of particular concern. Although this study confirms the presence rather than pathological outcomes, the proximity of MPs to reproductive tissues raises the possibility of endocrine disruption and impaired gamete development, warranting further histological investigation to assess implications for fisheries sustainability [53, 63, 64].

When compared with previously reported MP abundances in *L. rohita* and *P. pangasius*, typically ranging from 2.1 to 40.73 particles ind^−1^, the values observed in this study (58.27 ± 10.48 in *L. rohita* and 42.40 ± 5.40 in *P. pangasius*) are at the higher end or exceed earlier records. This suggests that freshwater systems in the Udupi region may be experiencing relatively elevated MP contamination, potentially due to increasing urbanization, wastewater input, and mismanaged plastic waste. The present study differs from most existing studies by evaluating five key organs: the gut, gills, liver, muscle, and gonads, rather than being limited to the gastrointestinal tract or gills alone. It also combines abundance and morphological data with polymer identification and risk‐based indices, thereby providing a more integrated understanding of MP exposure pathways and the potential health implications for both fish and humans.

### 3.3. MP Morphology, Color, and Size

**FIGURE 2:**
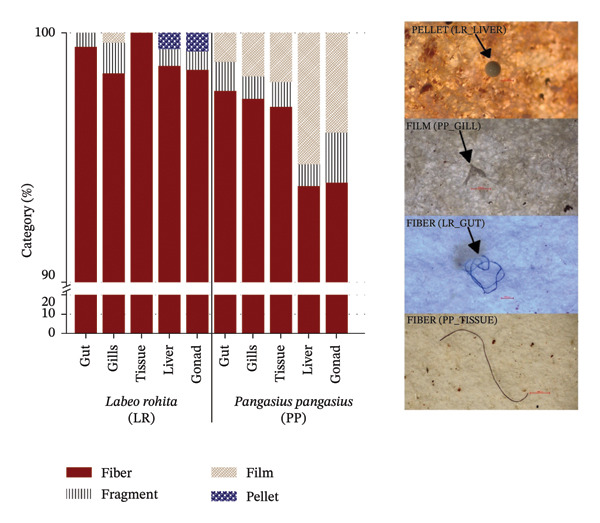
Stacked bar chart of MP shapes (fiber, film, and pellet) in LR and PP, with fibers being dominant. Representative microscopic images illustrate the typical morphology.

Morphological analysis revealed a clear dominance of fiber‐shaped MPs in both species. In *L. rohita*, fibers accounted for 98.97% of all particles, whereas in *P. pangasius*, they comprised 95.91%. Other morphotypes, such as fragments, films, and pellets, contributed only marginally to the total MP assemblage. In *L. rohita*, fragments represented 0.69%, pellets 0.23%, and films 0.11%, whereas in *P. pangasius*, films (2.83%) and fragments (1.26%) were slightly more represented but still minor relative to fibers (Figure 2; Supporting Table S3). This strong fiber dominance is consistent with observations from other freshwater and marine systems, where fibers have increasingly been recognized as a major MP form [45]. Notably, all the MPs identified in the muscle tissue of *L. rohita* were fibers, suggesting that fibrous particles may be more easily translocated into or retained within soft tissues than other shapes. Their slender geometry, flexibility, and tendency to entangle with biological structures may facilitate penetration or lodging in tissues, potentially leading to mechanical irritation or persistent low‐level inflammation [65].

The predominance of fibers is likely linked to their pervasive sources of origin. Synthetic textiles shed large numbers of fibers during washing, which are not fully retained by conventional wastewater treatment processes and can be transported into receiving waters [3, 47]. Additional sources include fishing gear, ropes, nets, and tire wear, all of which contribute to fibrous particles in aquatic environments [8, 38]. In contrast, fragments typically originate from the breakdown of rigid plastic items, such as packaging and consumer goods, whereas films largely derive from agricultural mulching materials and plastic bags [66–68]. The relatively low occurrence of fragments and films in the present study may reflect a lower local input of these specific plastic types, differential transport and settling behavior, or size‐related detection biases.

Color analysis revealed a wide variety of MP colors in both species, with a consistent dominance of blue and black particles. In *L. rohita*, 12 color categories were recorded, with blue (45.08%) and black (26.20%) being the most abundant, followed by red (11.67%), transparent (6.64%), green (5.26%), yellow (2.17%), and other colors (2.97%) (Supporting Table S4). *P. pangasius* showed a similar pattern across 10 color categories, with blue particles accounting for 60.53%, black for 18.71%, red for 9.91%, transparent for 7.23%, yellow for 1.26%, and other colors for 2.36%. The dominance of blue fibers is consistent with previous reports that have documented a high prevalence of blue synthetic fibers in aquatic biota and environmental samples [6, 53]. These patterns may partly reflect the spectrum of colors used in textiles and fishing gear but may also be influenced by the visual feeding behavior of fish. Many fish species rely on color cues when selecting prey; in turbid or low‐visibility conditions, brightly colored or contrasting particles may be mistaken for zooplankton or other food items, leading to selective ingestion.

**FIGURE 3:**
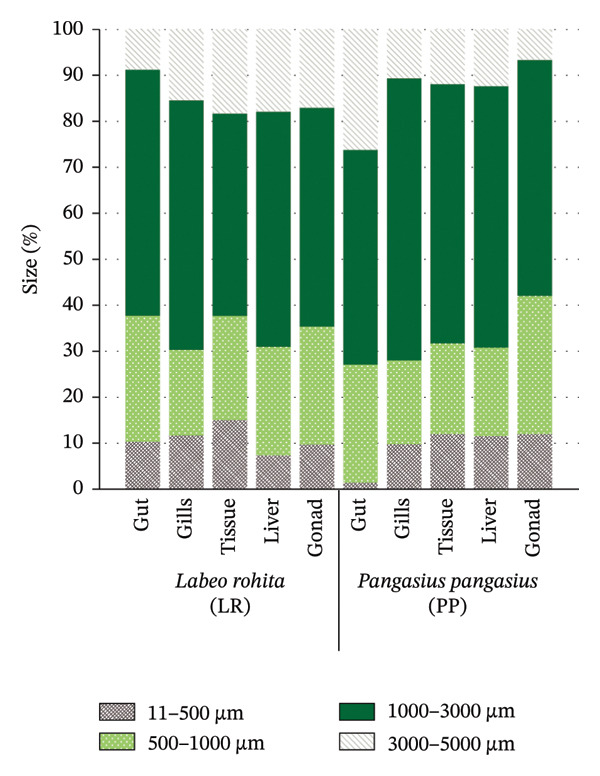
Size distribution of MPs across organs in the LR and PP groups. Particles measuring 1000–3000 μm were the most frequent in both species.

The size distribution of MPs indicated that most particles in both species fell within the 1000–3000‐μm class, which accounted for 50.92% of MPs in *L. rohita* and 54.09% in *P. pangasius*, respectively (Figure 3). Particles in the 500–1000‐μm range were the next most abundant (23.11% and 23.74%, respectively), followed by the largest size class (3000–5000 μm; 15.10% in *L. rohita* and 10.22% in *P. pangasius*) and the smallest class (11–500 μm; 10.87% and 11.95%, respectively). The relatively lower proportion of the smallest particles may be due to analytical limitations (e.g., filter pore size and visual detection thresholds) and possible loss during digestion and filtration. Biological factors may also play a role; larger particles may be more readily ingested along with food items or filtered water, particularly in herbivorous species interacting with macrophytes, periphyton, or sediments. In *P. pangasius*, the larger mouth size and omnivorous feeding strategy likely facilitate the ingestion of a broad range of particle sizes, whereas *L. rohita* may encounter and ingest particles that are entangled in plant material or detritus. The dominance of mid‐sized particles reflects a combination of environmental availability, hydrodynamic sorting, and size‐selective feeding.

### 3.4. Polymer Composition

FTIR–ATR analysis of the selected subset (*n* = 98) revealed distinct polymer signatures, indicating that *L. rohita* and *P. pangasius* are exposed to different suites of anthropogenic pollutants, shaped by their habitat preferences and regional economic activities. In *L. rohita*, the dominance of polyester (PES; 65.79%) serves as a molecular marker of domestic wastewater contamination. PES, a major component of synthetic textiles, is commonly released as microfibers during household laundering. Its high prevalence in the water column aligns well with the feeding ecology of *L. rohita*, a column to benthic feeder. In the Udupi–Mangalore region, where municipal wastewater treatment may be incomplete or bypassed through direct runoff into freshwater systems, these buoyant fibers remain highly bioavailable. The secondary occurrence of polystyrene (PS; 7.67%) further indicates inputs from the degradation of single‐use food packaging and expanded polystyrene (EPS) materials, which are widespread in urban environments [3, 69].

**FIGURE 4:**
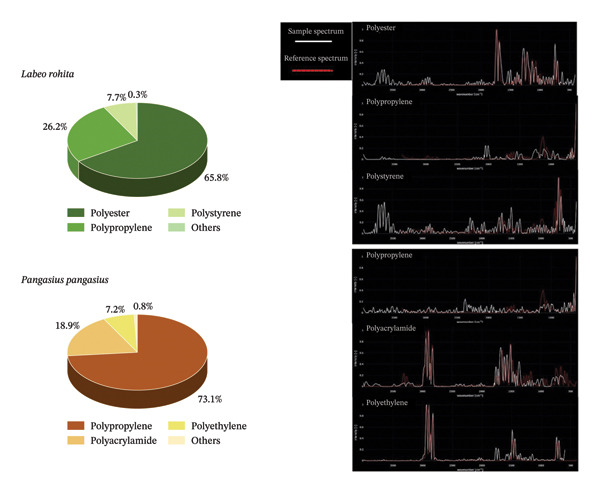
Pie charts of MP polymer types: polyester dominated in LR and polypropylene in PP. The representative FTIR spectra confirmed the polymer composition.

In contrast, *P. pangasius* exhibited a polymer profile dominated by polypropylene (PP; 73.11%). The intensity of fishing activities in coastal and inland waters of Karnataka likely contributes significantly to PP inputs through the mechanical degradation of ropes, nets, and aquaculture infrastructure. Unlike the textile‐derived fibers observed in *L. rohita*, the PP fraction in *P. pangasius* reflects a fishery footprint, where degraded materials either settle or remain suspended in deeper or near‐bottom waters, consistent with its feeding behavior [2, 70]. The detection of polyacrylamide (PAM; 18.87%) in *P. pangasius* is particularly noteworthy. PAM is widely used as a flocculant in industrial wastewater treatment and as a soil conditioner in periurban agriculture. Its presence suggests that *P. pangasius*, due to its omnivorous and more bottom‐oriented feeding strategy, integrates chemical signals from industrial effluents and agricultural runoff that are less pronounced in the upper water column inhabited by *L. rohita* [2, 70]. A small fraction of particles in both species (0.34% in *L. rohita* and 0.79% in *P. pangasius*) could not be confidently matched with reference spectra and were classified as “others.” These may represent weathered, mixed, or additive altered polymers with modified spectral signatures (Figure 4).

The contrasting polymer profiles, with PES dominance in *L. rohita* and PP and PAM dominance in *P. pangasius*, highlight that MP accumulation is not random but governed by the interplay between polymer properties such as density and buoyancy and species‐specific foraging behavior. While PES reflects diffuse municipal graywater inputs, PP and PAM indicate more localized, point source contributions linked to fisheries and industrial or agricultural activities. These findings have important implications for regional management and emphasize the need for a dual mitigation strategy targeting both household wastewater treatment and the management of end‐of‐life fishing gear.

### 3.5. Surface Characteristics and Elemental Composition

SEM imaging provides further insights into the environmental history and potential interactions of MPs with their surroundings. Fibers from both species generally exhibited elongated, thread‐like morphologies, with surface textures ranging from relatively smooth to moderately abraded. Grooves, scratches, and small surface irregularities are commonly observed, indicating mechanical wear during transport, resuspension, and ingestion [71, 72]. The fragments displayed more advanced degradation features, including deep cracks, pits, jagged edges, and embrittled surfaces. These attributes are consistent with prolonged exposure to UV radiation, hydrodynamic forces, and physical abrasion within the water column and sediment, as well as possible digestive processes in the gastrointestinal tract.

**FIGURE 5:**
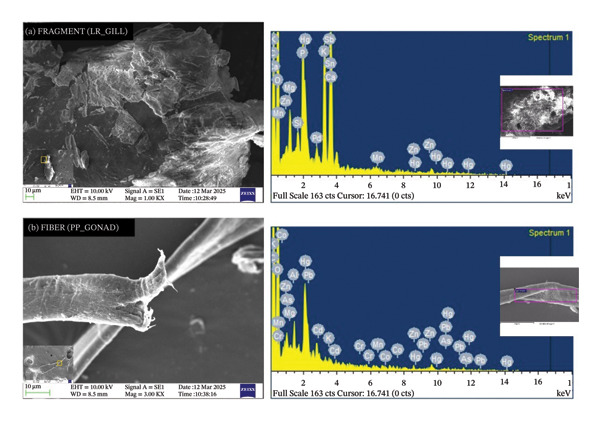
SEM images and EDS spectra of MP fibers from PP (top) and LR (bottom). SEM shows surface morphology; EDS indicates adsorbed metals (Pb, Hg, Cd, Cr, Zn, As). (a) FRAMENT (LR_GILL). (b) FIBER (PP_GONAD).

EDS analysis of selected fibers and fragments from the gills and gonads revealed the presence of multiple elements, including trace amounts of mercury (Hg) up to 0.16%, as well as measurable levels of lead (Pb), cadmium (Cd), zinc (Zn), chromium (Cr), copper (Cu), manganese (Mn), arsenic (As), cobalt (Co), tin (Sn), antimony (Sb), and palladium (Pd) (Figure 5). Many of these elements are associated with plastic additives (e.g., pigments, stabilizers, flame retardants) or environmental contamination from industrial, urban, or agricultural sources [44, 54, 57]. For instance, Fe and Cd are often present in inorganic pigments, whereas Cr is used in anticorrosive coatings for infrastructure. The co‐occurrence of these metals with MPs suggests that these particles may act as carriers or concentrators of toxic elements. However, SEM–EDS alone cannot distinguish whether the metals are bound to the plastic surface, embedded within additives, or reflect residues from the surrounding sediments or biofilms.

From a toxicological perspective, the combined presence of weathered MPs and associated metals in fish tissues can enhance the risk of oxidative stress, inflammation, and sublethal physiological effects. For example, MPs can provide sorption surfaces for metals and persistent organic pollutants, potentially increasing local exposure at the tissue level of the host. Nevertheless, the present data should be interpreted as qualitative indicators of co‐occurrence rather than as quantitative proof of MP‐mediated metal transport. Further geochemical analyses, including desorption experiments and metal partitioning studies, are needed to clarify the relative contributions of MPs and other environmental vectors to metal bioaccumulation.

### 3.6. MP Contamination and Associated Human Health Risks

The risk profile of MP ingestion is a multifaceted function of particle abundance, chemical composition, and physical morphology. The PHI revealed a marked contrast between the two species. While *L. rohita* fell into category IV (hazardous; PHI = 128.09), *P. pangasius* reached category V (extreme hazard; PHI = 139,925.10) (Supporting Table S5). This elevated value is primarily driven by the presence of PAM, which carries a high intrinsic toxicity score due to its monomer, acrylamide, being a known neurotoxin and carcinogen [46, 55, 73].

Furthermore, the CMPI, reflecting the dominance of fibers (0.99 for *L. rohita* and 0.97 for *P. pangasius*), indicates a high potential for mechanical injury. Fibrous MPs tend to have longer residence times within the gastrointestinal tract and can induce greater epithelial abrasion and inflammatory responses compared to spherical particles [67].

The EDI and EAI calculated in this study provide critical insights into the food safety implications of regional freshwater fisheries. To contextualize these findings, the results were benchmarked against recent national and global seafood contamination data (Table 1; Supporting Table S6). Based on species‐specific MP concentrations and a regional per capita fish consumption rate of 11.6 kg/year (31.8 g day^−1^), a consumer regularly consuming *L. rohita* and *P. pangasius* may ingest approximately 32.12 particles day^−1^ (11,724 particles year^−1^) and 19.40 particles day^−1^ (7081 particles year^−1^), respectively. When normalized to a 60‐kg adult BW, these values correspond to EDIs of 0.54 and 0.32 particles kg^−1^ BW day^−1^ and EAIs of 195.4 and 118 particles kg^−1^ BW year^−1^ for *L. rohita* and *P. pangasius*, respectively (Supporting Table S7).

**TABLE 1:** Comparison of estimated human microplastic intake from fish and shellfish with global studies.

Author (year)	Biota type (species)	Country	EDI (MP day^−1^)	EAI (MP year^−1^)
Devriese et al. (2015)	Prawn	Belgium	0.04–0.48	15–175 (14)
Karami et al. (2017)	Dried fish (*C. subviridis, J. belangerii, R. kanagurta, S. waitei)*	Bangladesh	0.7	246 (28)
Akoueson et al. (2020)	Fish (e.g., haddock, seabass)	UK	3.47–15.97	1267–5828 (2)
Saha et al. (2021)	Mussels, oysters, clams	India	22.15	8084.1 (62)
Wang et al. (2021)	Bivalves (clams, razor clams)	China	2.98	1088.64 (77)
Piyawardhana et al. (2022)	Dried fish	Sri Lanka	2.33–3.14	851–1147 (54)
Irnidayanti et al. (2023)	Green mussel (*Perna viridis*)	Indonesia	598.36	218,400 (24)
Mahu et al. (2023)	Fish (e.g., *Mugil cephalus*)	Nigeria	up to 490	up to 178,220 (42)
Mohsen et al. (2023)	Sea cucumber	China	0.078–5.1	28–1862 (47)
Guo et al. (2023)	Wild clam	China	177.58	64,816.21 (20)
Present study	*L. rohita and P. pangasius*	India	19.40–32.12	7081–11,724

These intake estimates exceed those reported for dried fish in Bangladesh (0.7 MPs day^−1^) [32] and Sri Lanka (2.33–3.14 MPs day^−1^) [74], as well as values reported for European finfish such as haddock and seabass (3.47–15.97 MPs day^−1^) [75]. The elevated intake observed in this study suggests that freshwater species in the Udupi region may accumulate higher MP loads than marine species from more open ocean systems. This pattern is likely influenced by dense urban and industrial discharges into relatively confined riverine catchments in Karnataka.

Although the estimated intake is lower than the extreme levels reported for filter‐feeding bivalves in Indonesia (593.36 MPs day^−1^) [76], it is comparable to high‐risk values reported for Indian shellfish (22.15 MPs day^−1^) [77]. Collectively, these findings indicate that regular consumption of freshwater fish in this region represents a significant and currently undermonitored pathway for human MP exposure.

These values should be interpreted as first‐tier conservative screening estimates. Future studies would benefit from larger and more diverse sample sizes, the inclusion of a wider range of commercially consumed species, and the consideration of regional and age‐specific fish consumption patterns. This would enable more accurate estimation of dietary exposure to MPs across different population groups. Despite the limitations, they fall within the range of magnitudes reported elsewhere for MP intake through seafood, highlighting that regular consumption of contaminated freshwater fish can represent a significant exposure pathway [47, 78].

### 3.7. Methodological Merits and Limitations

The methodology employed in this study was designed to maximize MP recovery while preserving polymer integrity. The use of a 10% KOH alkaline digestion protocol, supplemented with H_2_O_2_ for lipid‐rich tissues such as the liver and gonads, provided an effective balance between organic matter removal and the preservation of pH‐sensitive polymers, including polyester and polypropylene, which may degrade under strong acidic conditions. Furthermore, the application of Rose Bengal staining served as an important quality control measure to distinguish synthetic polymers from natural organic microfibers, thereby minimizing the risk of false positives.

However, certain methodological limitations should be acknowledged. The use of 11‐μm pore‐size filters establishes a lower detection threshold, and therefore, smaller MPs and nanoplastics may not have been captured in this assessment. In addition, although FTIR–ATR enabled reliable polymer identification, the manual handling involved in ATR analysis may bias characterization toward larger particles, typically greater than 50 μm. Future studies employing *μ*‐FTIR or Raman spectroscopy would enable more comprehensive detection and characterization of smaller MP fractions within complex tissue matrices.

## 4. Conclusion

This study presents the first comprehensive baseline assessment of MP contamination in freshwater fish from the Udupi region of Karnataka, providing critical insights into the extent, characteristics, and potential risks of MP exposure in two widely consumed species, *L. rohita* and *P. pangasius*. Across five key organs (gut, gills, muscle, liver, and gonads), 1510 MP particles were identified, with *L. rohita* showing the highest burden in the gills and *P. pangasius* in the gut. Fibers overwhelmingly dominated the MP assemblage (> 95%), with blue and black particles being the most frequently encountered particles. The mean MP abundance was substantially higher in *L. rohita* (58.27 ± 10.48 particles ind^−1^) than in *P. pangasius* (42.40 ± 5.40 particles ind^−1^), indicating species‐specific differences in exposure pathways, feeding strategies, and habitat interactions.

The polymer composition revealed further contrasts between the species. Polyester was most prevalent in *L. rohita*, consistent with textile‐derived inputs, whereas polypropylene dominated *P. pangasius*, reflecting the strong influence of fishing gear, aquaculture infrastructure, and local anthropogenic activities. Risk assessment indices, including the PHI, CMPI, and EDI, collectively indicated that the detected MPs may pose ecological risks to fish and potential health concerns for human consumers. The presence of weathered MPs exhibiting surface cracks, grooves, and abrasions, as confirmed through SEM analysis, suggests prolonged environmental exposure to the weathered MPs. Furthermore, the EDS detection of associated metals highlights the complex contaminant mixtures that freshwater organisms may encounter.

Of particular concern is the detection of MPs in internal organs, such as the liver and gonads, which points to possible translocation, bioaccumulation, and interference with reproductive physiology. While the presence of MPs in the gonads is a significant finding, it serves as an indicator of exposure and a potential risk factor rather than a confirmed biological effect. Such contamination may have long‐term implications for fish health, population dynamics, and food safety. These findings underscore the urgent need for improved plastic waste management, enhanced community‐level awareness, and targeted policy interventions to reduce MP inputs into freshwater environments. Protecting freshwater ecosystems and fisheries in the region is vital for ecological integrity and sustaining local food security and public health.

Future research should build upon these baseline findings by incorporating larger seasonally stratified sample sizes and expanding the analysis to additional freshwater species and habitats. Controlled laboratory exposure studies are essential for establishing dose–response relationships and disentangling the effects of MPs from those of co‐occurring pollutants. Integrating histopathological evaluation, oxidative stress biomarkers, reproductive endocrinology, and advanced omics technologies will help elucidate the mechanistic pathways underlying MP‐induced toxicity. Furthermore, investigating trophic transfer, biomagnification, and human dietary exposure will contribute to a more comprehensive understanding of the ecological and societal implications of MP pollution in freshwater ecosystems. Collectively, these efforts will enhance the scientific foundation needed to guide regulatory frameworks and mitigation strategies aimed at safeguarding aquatic resources and public health in India and beyond [79–82].

## Author Contributions

Kuruveetil Manikandan Ashitha: conceptualization, formal analysis, investigation, methodology, visualization, writing–review and editing. Anjali Tamrakar: conceptualization, formal analysis, investigation, methodology, writing–review and editing. Gopika Melethil: methodology, writing–review and editing. Anish Kumar Warrier: conceptualization, funding acquisition, project administration, resources, supervision, visualization, writing–original draft.

## Funding

Anish Kumar Warrier thanks the Anusandhan National Research Foundation (erstwhile SERB), Government of India, for a research project on microplastics (File No. CRG/2021/004725, dated June 24, 2022).

## Ethics Statement

The Institutional Animal Ethics Committee of MAHE is acknowledged for granting the necessary ethical clearance to carry out the research (Approval No: IAEC/KMC/36/2025, dated 25.02.2025).

## Consent

The authors have nothing to report.

## Conflicts of Interest

The authors declare no conflicts of interest.

## Supporting Information

Additional supporting information can be found online in the Supporting Information section.

## Supporting information


**Supporting Information** Table S1: Biological characteristics and morphometrics of *Labeo rohita* and *Pangasius pangasius*. Table S2: The organs examined, size range of specimens, microplastic abundance, color, and polymer types detected. Table S3: Size distribution and morphological types of microplastics detected in various organs of *Labeo rohita* and *Pangasius pangasius*. Table S4: Color‐wise distribution of microplastics in *Labeo rohita* and *Pangasius pangasius*. Table S5: Polymer composition and associated hazard assessment of microplastics detected in *Labeo rohita* and *Pangasius pangasius*. Table S6: Derivation of the daily fish ingestion rate used for exposure assessment. The table shows the conversion of annual per capita fish availability into a daily ingestion rate (31.8 g/day), which was applied in calculating the estimated daily intake (EDI) of microplastics. Table S7: Estimated daily intake (EDI) and estimated annual intake (EAI)

## Data Availability

Data generated in this study are included in the Supporting Information.
