# Supplementary material for: Organ‐Specific Microplastic Profiling and Polymer Characterization in Freshwater Fish Species of Karnataka
Source: J Toxicol. 2026 May 31;2026:8854199. doi: 10.1155/jt/8854199 (PMC13239186; doi:10.1155/jt/8854199)
Supplement: Supplementary file 1 — Supporting Information Table S1: Biological characteristics and morphometrics of Labeo rohita and Pangasius pangasius. Table S2: The organs examined, size range of specimens, microplastic abundance, color, and polymer types detected. Table S3: Size distribution and morphological types of microplastics detected in various organs of Labeo rohita and Pangasius pangasius. Table S4: Color‐wise distribution of microplastics in Labeo rohita and Pangasius pangasius. Table S5: Polymer composition and associated hazard assessment of microplastics detected in Labeo rohita and Pangasius pangasius. Table S6: Derivation of the daily fish ingestion rate used for exposure assessment. The table shows the conversion of annual per capita fish availability into a daily ingestion rate (31.8 g/day), which was applied in calculating the estimated daily intake (EDI) of microplastics. Table S7: Estimated daily intake (EDI) and estimated annual intake (EAI) [file JT-2026-8854199-s001.docx]

**Supplementary Information**

**Organ-Specific Microplastic Profiling and Polymer Characterization in Freshwater Fish Species of Karnataka**

Kuruveetil Manikandan Ashitha^1^, Anjali Tamrakar^2^, Gopika Melethil^3^, Anish Kumar Warrier^4^

**Supplementary Table S1: Biological characteristics and morphometrics of *Labeo rohita* and *Pangasius***

***pangasius***

| Species | *Labeo rohita* | *Pangasius Pangasius* |
| --- | --- | --- |
| Common name | Rohu | Panga |
| Family^⁎^ | Cyprinida | Pangasiidae |
| Habitat^⁎^ | Benthopelagic | Benthopelagic |
| Feeding Habit^⁎^ | Herbivores (grazing on aquatic plants) | Omnivorous (mainly animals (troph. 2.8 and up) |
| Feeds on^⁎^ | mainly plants/detritus | hunting macrofauna (predator) |
| Trophic level^⁎^ | 2.19 | 3.44 |
| Total length  (cm) | 41.63±1.55 | 42.37±2.14 |
| Total width  (cm) | 10.19±0.66 | 12.80±1.15 |
| Total Body Weight (Kg) | 0.86±0.10 | 1±0.15 |
| Mouth Length (mm) | 12.31± 3.91 | 32.61±2.29 |
| Mouth width(mm) | 11.99±4.04 | 17.90±3.95 |
| Gut weight (g) | 5.74±1.99 | 5.54±1.23 |
| Gill weight (g) | 5.76±2.55 | 4.81±1.06 |
| Tissue weight (g) | 10.08±0.64 | 10.44±0.28 |
| Liver weight (g) | 2.69±1.31 | 5.48±1.68 |
| Gonad weight(g) | 2.63±2.03 | 4.99±1.04 |
| Total organ weight (g) | 26.90±6.81 | 31.26±4.23 |
| ^⁎^Information was obtained from Froese, R., Pauly, D., 2019. FishBase. Available at <http://www.fishbase.org>  (accessed 10^th^ April 2025, 9:56 am) | | |

**Supplementary Table S2: The organs examined, size range of specimens, microplastic abundance, color, and polymer types detected.**

| Species | Region | n | Organ analysed | Frequency of occurrence (%) | Abundance  (MPs/ind) | Common type | Common colour | Common size | Detected Polymer | Reference |
| --- | --- | --- | --- | --- | --- | --- | --- | --- | --- | --- |
| *L. rohita* | Gujarat | 180 | GIT | 100% | 40.73±2.22  30.633±2.10  27.583±2.13 | Filament | Black | - | Polycarbonate  Polyurethane  PVC, PS, PAN etc. | [1] |
| *L. rohita* | Uttar Pradesh | 35 | GIT, Gills and Tissue | 100% | 4.17 ± 0.6 | Fiber | Pink and blue | 500-750μm | HDPE and LDPE | [4] |
| *L. rohita* | Bangladesh | 3 | GIT | 100% | - | Fibers | Transparent, red | 500 μm-1 mm | HDPE, PP/PE, EVA | [5] |
| *L. rohita* | Ganga river (Bihar) | 193 | GIT | 100% | 2.8 ± 1.9 | Fibers | White, blue, black | <5mm | Polyethylene, HDPE, LDPE | [2] |
| *L. rohita* | River ravi (Lahore, Punjab of Pakistan) | 36 total fish samples (3 species) | GIT, Gill, Muscles, Liver | 100% | 9.75 ± 6.65 | Fibers | - | - | PVC, PS, PP, PE | [7] |
| *L. rohita* | Chhota Nagpur zone of eastern India | 6 | GIT, GILL | 100% | 4.5 ± 1.7 | Fibers | Blue, black, red | <125 μm | PA, PE | [6] |
| *P. pangasius* | Dhanmondi Lake, Gulshan Lake, Hatir Jheel Lake, Bangladesh | 10 | GIT | - | 2.1 | Fragment,  Micro-pellet | Transparent | <100 μm | HDPE | [3] |
| *P. pangasius* | Chhota Nagpur zone of eastern India | 6 | GIT, GILL | 100% | 4.2 ± 1.8 | Fiber | Blue | 355–1000 μm | PVC, PE | [6] |
| *Pangasius hypophth-almus* | Malaysia | 5 | GIT | 100% | 4.00±3.16 | Film | Blue | 1000-5000 μm | - | [8] |
| *L. rohita and P. pangesius* | Karnataka, India (Present study) | 30 (15+15) | Gut, gills, tissue, gonad, liver | 100% | 58.27 ± 10.48 (*L. rohita*), 42.40 ± 5.40 (*P. pangasius*) | Fibers (97.68%), Films, Fragments, Pellets | Blue, black, red |  | Polypropylene, Polyester | Present study (2025) |

**Supplementary Table S3: Size distribution and morphological types of microplastics detected in various organs of *Labeo rohita* and *Pangasius pangasius***

|  | Organs | 11-500µm | 500-1000 µm | 1000-3000 µm | 3000-5000 µm | Fiber | Fragment | Film | Pellet |
| --- | --- | --- | --- | --- | --- | --- | --- | --- | --- |
| *Labeo rohita* | Gut | 19 | 50 | 99 | 16 | 183 | 1 | - | - |
|  | Gill | 29 | 46 | 135 | 38 | 244 | 3 | 1 | - |
|  | Tissue | 23 | 35 | 68 | 28 | 154 | - | - | - |
|  | Liver | 11 | 36 | 78 | 27 | 150 | 1 | - | 1 |
|  | Gonad | 13 | 35 | 65 | 23 | 134 | 1 | - | 1 |
|  | Total | 95 | 202 | 445 | 132 | 865 | 6 | 1 | 2 |
|  | Percentage | 10.87% | 23.11% | 50.92% | 15.10% | 98.97% | 0.69% | 0.11% | 0.23% |
| *Pangasius pangasius* | Gut | 25 | 47 | 83 | 20 | 171 | 2 | 2 | - |
|  | Gill | 10 | 22 | 65 | 12 | 106 | 1 | 2 | - |
|  | Tissue | 12 | 19 | 58 | 11 | 97 | 1 | 2 | - |
|  | Liver | 12 | 21 | 63 | 12 | 101 | 1 | 6 | - |
|  | Gonad | 17 | 42 | 75 | 10 | 135 | 3 | 6 | - |
|  | Total | 76 | 151 | 344 | 65 | 610 | 8 | 18 |  |
|  | Percentage | 11.95% | 23.74% | 54.09% | 10.22% | 95.91% | 1.26% | 2.83% |  |

| *Labeo rohita* | Blue | 394 | 45.08 |
| --- | --- | --- | --- |
|  | Black | 229 | 26.20 |
|  | Red | 102 | 11.67 |
|  | Green | 46 | 5.26 |
|  | Transparent | 58 | .64 |
|  | Yellow | 19 | 2.17 |
|  | Pink | 9 | 1.03 |
|  | Violet | 3 | 0.34 |
|  | Brown | 4 | 0.46 |
|  | White | 1 | 0.11 |
|  | Orange | 2 | 0.23 |
|  | Purple | 7 | 0.80 |
| *Pangasius pangasius* | Blue | 385 | 60.53 |
|  | Black | 119 | 18.71 |
|  | Red | 63 | 9.91 |
|  | Transparent | 46 | 7.23 |
|  | Orange | 3 | 0.47 |
|  | Green | 5 | 0.79 |
|  | Yellow | 8 | 1.26 |
|  | Pink | 2 | 0.31 |
|  | Violet | 3 | 0.47 |
|  | Brown | 2 | 0.31 |

**Supplementary table S4: Color-wise distribution of microplastics in *Labeo rohita* and *Pangasius pangasius***

**Supplementary Table S5: Polymer composition and associated hazard assessment of microplastics detected in *Labeo rohita* and *Pangasius pangasius***

| Species | Polymer | Monomer | Score | PHI | Hazard  category | Risk category |
| --- | --- | --- | --- | --- | --- | --- |
| *Labeo rohita* | Polypropylene  Polystyrene  Polyester | Propylene  Styrene  Ester | 1  30  - | 26.20  7.67  - | III  IV  - | High  Danger |
| *Pangasius pangasius* | Polypropylene  Polyacrylamide  Polyethylene | Propylene  Acrylamide  Ethylene | 1  22240  11 | 73.11  419622.64  79.56 | III  V  III | High  Extreme danger  High |

**Supplementary Table S6: Derivation of the daily fish ingestion rate used for exposure assessment. The table shows the conversion of annual per capita fish availability into a daily ingestion rate (31.8 g/day), which was applied in calculating the Estimated Daily Intake (EDI) of microplastics.**

| Convert Annual Availability to Daily Ingestion Rate (IR): | |
| --- | --- |
| - Annual per capita fish availability | 11.6 Kg/year |
| - Daily Ingestion Rate (IR): | $= \frac{11.6 kg/Year}{365\frac{days}{year}}$= 0.0318 Kg/day, 31.8g/day |

**Supplementary Table S7: Estimated Daily Intake (EDI) and Estimated Annual Intake (EAI)**

| Fish Species | MP concentration in muscle (particles/gm) | Daily Fish Ingestion Rate (g/day) | Estimated Daily Intake (EDI) - particles/day | EDI (particles/kg-bw/day) | Estimated Annual Intake (particles/year) | (EAI)-particles/kg-bw/year |
| --- | --- | --- | --- | --- | --- | --- |
| *L. rohita* | 1.01 | 31.8 | 32.12 | 0.54 | 11,724 | 195.4 |
| *P. pangasius* | 0.61 | 31.8 | 19.40 | 0.32 | 7081 | 118.0 |

Notes-

- Adult body weight (BW) for normalisation is assumed to be 60 kg.
- EDI was first calculated as particles per day and then normalised per kilogram body weight.
- EAI was calculated by multiplying EDI by 365 days.

**References**

[1] Jaydipsinh C, Hitesh C, Ali M, George LB (2025) Analysis of ingested microplastics by the selected freshwater fish. Pollution 11(2):485–496. https://doi.org/10.22059/poll.2024.381322.2532

[2] Kumari N, Yadav DK, Khan PK, Kumar R (2023) Occurrence of plastics and their characterization in wild caught fish species (*Labeo rohita*, *Wallago attu* and *Mystus tengara*) of River Ganga (India) compared to a commercially cultured species (*L. rohita*). Environ Pollut 334:122141. https://doi.org/10.1016/j.envpol.2023.122141

[3] Mercy FT, Alam AR, Akbor MA (2023) Abundance and characteristics of microplastics in major urban lakes of Dhaka, Bangladesh. Heliyon 9(4):e14587. https://doi.org/10.1016/j.heliyon.2023.e14587

[4] Pandey N, Verma R, Patnaik S, Anbumani S (2023) Abundance, characteristics, and risk assessment of microplastics in indigenous freshwater fishes of India. Environ Res 218:115011. https://doi.org/10.1016/j.envres.2022.115011

[5] Parvin F, Jannat S, Tareq SM (2021) Abundance, characteristics and variation of microplastics in different freshwater fish species from Bangladesh. Sci Total Environ 784:147137. https://doi.org/10.1016/j.scitotenv.2021.147137

[6] Patidar K, Alluhayb AH, Younis AM, Dumka UC, Ambade B (2024) Investigation of microplastic contamination in the gastrointestinal tract of fish: A comparative study of various freshwater species. Phys Chem Earth A/B/C 136:103760. https://doi.org/10.1016/j.pce.2024.103760

[7] Raza MH, Jabeen F, Ikram S, Zafar S (2023) Characterization and implication of microplastics on riverine population of the River Ravi, Lahore, Pakistan. Environ Sci Pollut Res 30(3):6828–6848. https://doi.org/10.1007/s11356-022-22440-yk

[8] Sarijan S, Azman S, Mohd Said MI, Lee MH (2019) Ingestion of microplastics by commercial fish in Skudai River, Malaysia. EnvironmentAsia 12(3). https://doi.org/10.14456/ea.2019.47
